# Selections from Foreign Journals

**Published:** 1889-04

**Authors:** 


### SELECTIONS FROM FOREIGN JOURNALS.

### TSCHAK—AN EPIDEMIC DIARRHŒA IN CAMELS.

Abstracted from "Archive of Veterinary Science, St. Petersburg, Russia." By Robert Formad, V. M. D., Assistant Demonstrator of Normal Histology, University of Pennsylvania.

Dr. Vedernikoff, Veterinarian to the Kirgeese herds in the government of Astrachan-Russia, in a scholarly paper of which the following is an abstract, describes a very interesting disease in camels, which he ascribes to the action of sea water and the influence of climatic conditions. It appears to be from his description a disease of epidemic character, of unique type.

Epidemic diarrhœa of Camels, known in the Kirgeese language by the name of "Tschak," is a disease which has developed only within the last twenty years in the seacoast valleys of the Caspian Sea, especially in some districts of the departments of Astrakhan.

Since the time that pastures have changed in consequence of quicksand invading the luxuriant flatlands, the disease has developed more and more frequently. Before the year 1870, isolated cases only have been seen, when heavy rains followed prolonged dryness. The rain water having collected in pools, becoming stagnated, and when used for watering the animals, produced the disease in feeble and young camels. On these flatlands of the Caspian Sea, which became overflowed by sea-water, (driven on by the south-east winds during the autumn), vegetation became profuse after the subsidence of the water. This grass had also been noticed to produce the disease. The epidemic character had not been observed until 1881–82, when the disease occurred constantly, without any marked cause, taking often an enzootic character. In 1885, it reached such an extent, that entire herds of camels were destroyed, and, strangely to say, that young and feeble, as well as old and robust animals, contracted the disease in the same degree. Further, sometimes the mother was perfectly well, while the suckling young became sick. In some herds more adults became sick, in other herds the younger animals, while in still others the former as well as the latter contracted equally the disease. The percentage of recovery differed also very greatly from five to seventy per cent., while instances of loss of the total herd have occurred. The rate of sickness among the camels of different owners, was various, although the animals were under the same conditions of food, drinking water and pasture. For instance, one owner had a large number of animals becoming sick, the other only a few or none ; so was also the percentage of recovery. Sometimes the herd which had the largest number of sick animals did not loose any, while the herd with the least number of sick, lost every one affected. Considering the conditions under which the disease originates, the etiological factors must be the grass, which had been submerged by sea-water rich in chlorine, sulphuric-iodine and bromine compounds, or had grown from soil saturated with those salts ; or it must be the drinking of the sea-water itself, or of rain-water, which, during long standing, had become saturated with these compounds. To the action of these substances the reporter attributes the cause of the disease, which is simply a severe catarrhal condition of the gastro intestinal tract.

The chlorides of potassium and of sodium of which the waters of the Caspian Sea contain a very large quantity, are not decomposed in the stomach, but irritate the mucous membrane of the alimentary tract. Acting as absorbents of liquid they produce beside increased peristaltic action and increased secretion of mucous and water. The action of sodium chloride on the blood aids in formation of blood corpsucles, while the action of potassium chloride leads to destruction of the same. The interchange of nitrogenous substances is diminished in the presence of excess of salt, and if the excess is constant it produces derangement of digestion. The blood becomes less coagulable, and sodium chloride increases the blood pressure, in consequence of which the pulse becomes slow, the action of the heart irregular, with diminution of bodily temperature and slowing of respiration. Upon the nervous system the chlorine compounds produce weakness, diminished sensibility and sluggishness in motion. In the excreting glands (pancreas and kidneys), they produce irritation and increased functional activity. The sulphur compounds act similarly to the chlorine compounds being only weaker in their action. The bromine compounds produce a burning pain and irritation of the mucous membrane of the stomach and intestines. In small doses they excite the vascular system and heart, but with diminution of the pulse rate and temperature. These compounds act also depressent on the nervous system, producing sleepiness, giddiness, etc.; and produce, similar to the chlorine compounds, increased functional activity of the excreting glands. The iodine compounds are more powerful in their action than the bromine and produce intense irritation of the stomach and intestine, deep congestion, swelling of the mucous membrane and dilatation of the peripheral vessels of the stomach. The respiration is at times increased, at other times diminished; temperature is increased. The action of the iodine compounds on the nervous system is similar to that of the bromine compounds, but much weaker. The excreating glands are also irritated and the secretion increased.

The disease in the camels seems to develop without any prodromic symptoms. An animal may be taking food and water, and work willingly one day, becoming the following day affected. Three distinct stages can be observed in which we find diarrhœa present in different degrees. The first stage lasts for twenty days, during which time the symptoms appear in a mild degree. The bowels become slightly loose, the excrements are passed in balls, which are very friable, moist, of a greenish color and characterestic odor, the latter very faint in the beginning. The general appearance of the animal is very little changed. Eating as well as rumination are normal with slight noticeable thirst. There is a little discharge from the eyes and a redness of the conjunctiva, but the latter is not marked and may not be noticed by an inexperienced eye. The respirations become increased to 20° per minute. The pulse 40 to 50 per minute. The temperature 38° C.

The second stage lasts about nine days, and the above mentioned symptoms become more intense. The excrements become now semiliquid of a grayish-green or light-brown color. The discharge from the eyes become greatly increased and contains considerable amount of pus, which gives it a whitish color. The conjunctiva becomes bright red; respirations run up to 25° per minute, pulse to 70° per minute, becoming irregular, and the temperature reaches 40° C. The appetite and rumination becomes almost entirely lost, the thirst increased. The animal is dull, lies down frequently, turning his head backward, grunting at the same time, and after having lain down once rises with great difficulty, every motion becomes slow, and the general condition of the animal expresses a low disordered state.

Third stage lasts only five days, and differs from the second by greater intensity of the symptoms. The excrements are now perfectly liquid of a dark color, mixed with blood, and of an offensive odor. The mucous membrane of the rectum is deeply injected and protrudes. The discharge from the eyes is of a thicker consistency, milk-white in color, and glues the eyelids together, while the orbital vessels are much congested. Respiration is reduced to 20° per minute ; pulse to 40° per minute ; temperature to 37° C. The mouth becomes filled with foaming saliva. The appetite, rumination and desire for water is almost entirely lost. The animal becomes very feeble, lies down in a drowsy condition and grunts constantly. Weak or young animals become comatose from the third or fourth day. According to the statement of the natives, recovery, if it takes place, usually occurs between the first and second stages ; the looseness of the bowels lasts for six weeks, and we have a general amelioration of the symptoms ; recovery between the second and third stages lasts not more than two weeks. Perfect recovery however is very slow, and two or three months may pass before the animal regains perfect health. The most marked sign of returning health is the change of the excrement from the liquid diarrhœic state, first, to partially formed brittle balls, which fuse together and are covered with an opaque layer, often accompanied with blood ; then into roundish shaped balls of a grayish-green color, which is the natural excrement of the animal. At the same time that the excrement is changed in character we find the conjunctiva becoming paler, its discharge diminishing and of a serous character. The respiration and pulse become slower, temperature becomes normal, and the animal returns to its natural appetite and rumination. The general condition of the animal improves, but the adipose tissue of the humps return only a long time after. At frequent intervals the animal grunts indicating pain, for a long time after amelioration of the symptoms have taken place.

POST-MORTEM APPERANCE.

1. *External appearance.*—No lesion or changes could be seen in the skin ; body emaciated and perfect absence of fat in the humps ; eyes covered with a discharge rich in pus ; on opening the eyelids the conjunctiva was deeply congested, and the pupils dilated ; below the eyes the hairs were wetted with lachrymal discharge mixed with pus ; from the nose a clear mucous discharge was running ; the mucous membrane of the mouth was anæmic ; hind quarters stained with gray colored excrement in which partly digestive food was seen, and conveying a sharp pungent odor ; the rectum partly protruding, showing deep congestion of the mucous membrane. The skin after removal pale on its inner side, while the larger vessels were filled with blood ; the muscles were also pale in color.

2. *Abdominal cavity.*—The large and small omentum presented complete absence of fat and great hyperæmia ; the mesentery congested, and the larger vessels engorged with blood. The serous membrane of the stomach and intestines deeply congested with great distention of the larger blood-vessels which present a cord-like appearance. The mucous membrane was just as congested as the serous membrane. The walls of the third and fourth stomachs as well as the rectum were very much thickened, while in other parts of the intestines the thickness was much less marked. The contents of the first stomach consisted of partly chewed food. The contents of the second and third stomach was liquid, of a brownish or gray-green color with the specific pungent odor, while that of the intestine was of the same consistency and color, but of a more offensive odor. The capsule covering the liver was light in color. The liver of normal size and density showed a marble coloration, which had been produced by darker and lighter spots. On section the interior of the liver presented similar appearance. The gall-bladder distended by dark-green bile, thick in consistency. Spleen was enlarged one and a half times ; filled with blood, soft, and the splenic pulp could be scraped easily ; the capsule of a dark-violet color. The pancreas was enlarged, of a pale rose-color, and showing most marked the borders of of the individual lobules. The kidneys were without change in shape or size but their capsules adherent. On section the cortical layer was dark, while the medullary layer of a much lighter color, still the whole organ congested. The ureters partly thickened and the mucous as well as the serous membranes congested. The bladder greatly distended with urine of a light yellow color, and by allowing it to stand yielded a flaky whitish precipitate. The entire abdominal cavity was greatly congested, particularly all the larger vessels were over-distended by blood and protruding prominently from these tissues.

3. *Thoracic cavity.*—The diaphragm was very congested in its tendinous portion, while the muscular portion was very pale, pleura somewhat congested, but not as deep as the pericardium. Both auricles and ventricles of the heart were filled with partly liquid blood in which clots of larger and smaller size floated. The lungs were congested and likewise the bronchials. The larger thoracic and abdominal vessels contained semi-liquid blood, dark in color. The cranial cavity, dura mater and pia, as well as the substance of the brain did not present microscopically any abnormal changes.

---

THE THEORIES AND TREATMENT OF SNAKE-BITE IN THE AUSTRALIAN COLONIES.—Dr. Creed says : From the experience which I have had of cases of snake-bite in New South Wales, and from inquiries I have made as to the particulars of others which have occurred in the practice of my friends who have kindly given me an account of them, I have been obliged to arrive at the conclusion that, with the exception of the species of *Hoplocephalus*, especially the "large-scaled snake," or, as it is called in Tasmania, the Diamond snake (*Hoplocephalus superbus*), and the brown banded or tiger snake (*Hoplocephalus curtus*), the death adder (*Acanthophisantaretica*), the brown snake (*Diemenia superciliosa*), and a large and vigorous specimen of the black snake (*Pseudechis porphyriacus*), the danger of death from the effects of the bite of the numerous venomous snakes common to the colony is very much exaggerated, and that in the majority of cases showing serious symptoms, these symptoms are really the effect of intense fear and not of snake poison.

The essential object to be attained in the treatment of every bite from a venomous snake is the prevention of the absorption of the poison, and this can be done only by the stopping of the circulation as quickly as possible between the bite and the heart, and the prompt and efficient excision of the punctured part to such a depth as shall insure either the absolute removal of the injected poison, or at all events, its exposure to such an extent that it can easily be removed by washing with the nearest attainable fluid, preferably water. He strongly insists on the advantages of the excision of, instead of incision into and about, the puncture caused by the teeth of venomous snakes, for the following reasons: Excision of the very lowest point of the puncture is easily practicable by any intelligent person, without danger of causing more serious hemorrhage than can easily be controlled by the application of a pad. If done in this way the whole of the poison will be removed. On the other hand, incision made into and about the wound may or may not remove the venom, which might easily be retained at the original spot into which it was injected by the bite, if the cut made did not actually enter into and freely expose it. In addition, the danger from hemorrhage would be very much greater from the wound made by the incision, as the point of the knife would probably enter to a greater depth than the wound made in excision. In fact, unless the operator has a fair knowledge of anatomy, he might easily in many places wound an artery lying comparatively superficially, and cause death by hemorrhage in a few minutes.

With regard to sucking the wound, the entire removal of the poison by its means is problematical, and such treatment is not without danger to the person sucking. Unless made by a person with some knowledge of anatomy, he thinks the incision should be made by pinching up the parts to be cut away, and then removing them with the middle of the blade, and not by inserting the point of the knife and cutting round the bite. The wound should be well washed and moderate bleeding encouraged. It would be well to wash it with a weak solution of permanganate of potash, or it might have an application of strong nitric or carbolic acid, or the actual cautery. The permanganate is probably an antidote if brought in direct contact with the poison, but when thrown into the general circulation it can be only of little if any use, for it will attack not only the snake poison but will oxidize all organic matter with which it comes in contact. After the ligature is removed the patient should be carefully watched and symptoms treated, the best stimulant being ether under the skin. Compulsory exertion or walking the patient about is forbidden. Dr. Creed uses alcohol for the treatment of the symptoms of exhaustion but not as a special treatment of the poison.

By experiments made by Mr. Krefft, in Sydney, it has been demonstrated that no effect follows the infliction of a bite by one of the most venomous snakes either on itself or on another venomous snake of the same or a somewhat similar class. From this Dr. Creed argues that if physiological antidote is to be discovered, it must be sought in this peculiar property inherent in the snake, which can hardly be merely consequent on structure, but may be the result of some substance diffused in the snake itself, which diligent research may demonstrate to be capable of isolation, and, if so, of use in the treatment of snake-bite. Dr. Creed is of the opinion that in order to prevent fear from acting harmfully on the patient he would when needed use ether as an anæsthetic. He would keep the patient barely unconscious for an hour or two, then allow him to recover enough to judge of his condition, watching pulse and respiration all the time carefully. If necessary he would bring the patient again and again under the influence of the ether, so long as might be required.

An interesting article on snake-bite and its treatment was read recently by Dr. Augustus Mueller before the Victorian Medical Society.

The doctor's theory is that the poison of the snake does not act as a special blood poison ; all the symptoms are due to deficient nerve action. The insidious venom travels quickly to the nerve centres, affecting in rapid succession the anterior column of the cord, the vaso-motor centers of the brain, and causing paresis and paralysis of the muscles of the lower extremities, accompanied by rapidly diminished force of heart action and blood pressure, and in a very short time by sleep, which in severe cases deepens into coma ; the latter sometimes remains till death, but more frequently passes off again, even in fatal cases. The usual method of death is by syncope. Dr. Mueller then made some interesting comparisons between the effects of the poison of the Australian and the Indian snakes. Both are without doubt nerve poisons. The Australian snake-poison seems more diffusible and develops its effects more quickly, and is distributed uniformly over the whole motor system. It is on this account, and also on account of the smaller quantity of poison at the disposal of Australian snakes that their bite is less frequently fatal than those of the Indian snakes of the East Indies.

The poison of the latter snakes, especially of the cobra, seems less diffusible, and takes a longer time as a rule to develop its effects ; but once fully developed the course is much more quickly fatal, owing to the fact that the poison concentrates its action on special nerve centers, instead of being equally distributed over all. Dr. Mueller has had thirty years practice in a district in which snakes abound, and he has unusual opportunities of studying the effects of snake-bite, both on men and the lower animals. He tells how he came to have his attention called to the use of the antidote that he now so strongly recommends, viz., strychnia. Strange to say it was in a case of spider-bite in a child two years old. He asserts that effect of spider-bite differs only in degree from that of snake-bite. Incidentally it may be mentioned here that there are a number of fatal cases of spider-bite on record in the Colonies ; in Queensland they are not at all of very rare occurrence. In commenting on this case the doctor says he was at once struck by the close resemblance, if not identity, of symptoms here present with those of snake-bite—loss of power in the legs, extreme pallor, coldness of skin, feeble heart action, with pulse scarcely perceptible at the wrist, dilated pupils insensible to light, and inability to move either arms or legs.

Treatment was begun with the usual orthodox remedies—eliminative, stimulating, alkaline, etc., etc., but all failed. The collapse increased rapidly, respiration became difficult, eyes glassy, heart's action barely perceptible, pulse gone at the wrist. In short, the patient would die in a few moments to all appearances. Now came to me a happy thought, says the doctor; like a flash it passed through my mind that the child's inability to walk soon after being bitten might have been caused by the subtle poison exerting a specific depressing effect on the motor cells in the anterior columns, gradually extending over the rest of the motor and also the vaso-motor centers. All the ominous symptoms thus found a ready explanation, and strychnia suggested itself as the remedy for this condition of things. To use his own words, "I hastily procured some strychnia, and injected ten minims of the official solution into the arm. In a half hour's time my little patient was sitting up, snatched, I have reason to believe, from the very jaws of death." He then relates a case in which he used this remedy in a venomous snake bite, with equally good success. In another case he produced, through the agency of the strychnia, a marvellous change in a desperate case in less than an hour, watched the patient for a number of hours and then gave his sanction to his going home. The lad felt strong and entirely well, mounted his horse and rode off; but he died next morning before the doctor could reach him. "I did not know then, as I do now, that even strychnia is more quickly decomposed and thrown out of the system than the snake-bite poison and that the latter will lurk about in the holes and corners of the system and resume its work of destruction as soon as the antagonist is removed that kept it in check. I had injected into the system of a mere boy, within half an hour not less than one-third of a grain of strychnia; but even this comparatively large quantity was evidently only just enough to counteract for a time, and as long as it lasted, the deadly venom. If I had only dared to push the use of the drug a little further, until it produced the usual symptoms in a moderate degree; if I had only directed it to be taken internally, from time to time in ordinary doses as I do now, after the painful lesson this case gave me, I have not the remotest doubt, from what I have seen of its action since, that the life of this lad would have been saved."—*Medical and Surgical Reporter.*

---

THE ACTION OF VARIOUS OILS ON HEALTHY ANIMALS.—According to the Paris correspondent of the *British Medical Jour-*

*nal*, December 15, 1888, M. Daremberg has been making a series of experiments on healthy animals, with the view of ascertaining the effect of different oils on tuberculous animals. He found that different oils used as food, and cod-liver oil, may be taken for three months and a half without evil effects by guinea-pigs in daily doses of one cubic centimetre, and by rabbits in doses of three to four cubic centimetres. The oil is easily administered through a glass tube placed in the animal's mouth; the animal usually sucks the tube with avidity. M. Daremberg gave subcutaneous injections of a quarter, half, or one cubic centimetre of oil every day, every second day, or every four days, into the abdomen, the back, or the paws of guinea-pigs and rabbits. The oil and instruments were previously sterilized. In no case were abscesses observed in the animals, but the injections produced the following results: Nearly all the guinea-pigs died after a varying interval of time; they all presented a similar lesion,—namely, fatty perisplenitis. Around the spleen there was a collection of yellowish fat, soluble in ether. The spleen adhered to the kidney and stomach; the stomach adhered to the liver and the parietal peritoneum. Fatty patches were detected on the liver; in some cases the biliary vesicle was completely surrounded by these yellowish masses. In every case there was a yellowish track under the peritoneum, below the costal insertions of the diaphragm on the left, and sometimes on the right side. In no case did the liver adhere to the parietal peritoneum on the right side of the abdomen. The fatty infiltration almost entirely disappeared when the injections were suspended for a certain time. But the adhesions persisted, as M. Daremberg ascertained by examining several animals which were killed. He found these adhesions: First, in a guinea-pig to which eight and a half cubic centimetres of white cod-liver oil had been given in eighteen days, and which was killed forty-two days after the last injection. Secondly, in a guinea-pig to which seven cubic centimetres of pale cod-liver oil had been given in sixteen days, and which was killed fifty-two days after the last injection. Thirdly, in a guinea-pig to which eight cubic centimetres of brown cod-liver oil had been given in sixteen days, and which was killed sixteen days after the last injection. Fourthly, in a guinea-pig to which one cubic centimetre of oleic acid (chemically pure) had been given, and which was killed forty-three days after the injection. The amount of oil required to cause death in guinea-pigs varied considerably. One guinea-pig died eight days after receiving four cubic centimetres of white cod-liver oil; another died twenty-three days after two cubic centimetres and a half; a third, which received eighteen cubic centimetres in thirty-six days, survived; it was killed thirty days after the last injection, and was found to present the adhesions in the peritoneum above described. One cubic centimetre of pale cod-liver oil killed one rabbit in six days; five cubic centimetres killed another in thirteen days; twenty-five cubic centimetres killed a third in fifty-two days. Two rabbits, to which six cubic centimetres and a half of brown cod-liver oil had been administered, died in fifteen days; one to which six cubic centimetres had been given died in twenty-five days; another died, after receiving nine cubic centimetres, in thirty-six days; another, after two cubic centimetres and a half, in six days; another, after four cubic centimetres, in eleven days; another, after six cubic centimetres and a half, in twenty-two days; another, after seven cubic centimetres and a half, in twelve days; another, after four cubic centimetres and a half, in thirty-seven days; and another, after twelve cubic centimetres, in twenty-eight days. A guinea-pig, to which one cubic centimetre of oleic acid, as usually sold, was given, died the same day; another, which received one cubic centimetre and a quarter, died in three days; two others, which received one cubic centimetre, died in four days; another died in fifteen days, after receiving one cubic centimetre; another in seventeen days, after one cubic centimetre and a quarter; another in nine days, after one cubic centimetre; another in eight days, after four cubic centimetres; and another in six days, after four cubic centimetres. One guinea-pig died in seven days, after receiving one cubic centimetre of olive oil; another died in thirteen days, after receiving one cubic centimetre and a half of colza oil; another in thirty-one days, after four cubic centimetres of cotton oil. One died in forty-seven days, after receiving six cubic centimetres of oil of *Fagus sylvatica*; and another died after receiving three-quarters of a cubic centimetre of crude petroleum oil. In the case of rabbits the effects of subcutaneous injections of oil were considerably less severe. After receiving half a cubic centimetre of oil every day for a month these animals were killed. They all presented the fatty lesions and adhesions observed in the guinea-pigs, but in a minor degree; and they appeared to be perfectly healthy. In every case there were oily tracks in the peritoneum, around the liver, kidney, stomach, spleen, and costal insertions of the diaphragm, but there were no adhesions in these organs. The stomach alone was attached by somewhat loose bands to the parietal peritoneum. A few patches of fat were found on the liver and spleen. The fatty infiltration of the large omentum gave it the aspect of a loosely-woven net. The rabbits which received similar doses of crude petroleum oil presented abscesses at the spots where the inoculations were made, and the same fatty lesions, rendered more apparent by the black color of the oily tracks. Some black spots were also noticed on the lungs. Most of these animals were in good condition at the time of their death. Several of them increased in weight during the period in which the experiments were made.—*Therapeutic Gazette.*

